# Disarticulation methods in orthodontic treatment - An update

**DOI:** 10.6026/973206300213905

**Published:** 2025-10-31

**Authors:** Suchareeta Panda, Nitu Gautam, Saibalini Pani1, Subhrajeet Narayan Sahoo, Sumita Mishra, Krishna S, Miral Mehta

**Affiliations:** 1 Department of Orthodontics, Institute of Dental Sciences, Siksha 'o' Anusandhan (Deemed to be University), Bhubaneswar, Odisha, India; 2 Department of Pediatric and Preventive Dentistry, Karnavati School of Dentistry, Karnavati University, Gandhinagar, Gujarat, India

**Keywords:** Disarticulation, bite raisers, bite planes, orthodontic auxiliaries, deep bite correction, crossbite management

## Abstract

Disarticulation methods, commonly referred to as bite raisers or bite planes, play a pivotal role in orthodontics. This review consolidates current literature on the various designs, classifications and modifications of anterior and posterior bite planes, along with their clinical indications, ideal requirements and associated complications. Special emphasis is placed on innovations that improve patient comfort, reduce dependence on compliance and minimize adverse effects such as trauma, speech interference, or undesired tooth movement. The article further highlights the significance of tailoring bite raiser selection to the patient"s facial type, malocclusion, mandibular plane angle and treatment mechanics. A comprehensive understanding of the biomechanics, application protocols and potential risks is essential for optimizing treatment outcomes.

## Background:

When treating malocclusions like angles class II div 2, scissors bite deep bite and anterior cross bite, it is common practice to temporarily elevate the bite to clear the space between the teeth, improve the discrepancy between the crowns and the bite and create room for the maxillary and mandibular incisors to move freely and effectively. This, in turn, prevents orthodontic brackets from breaking while treatment is underway [1, 2-3]. As far as clinical orthodontists are concerned, raising the bite is nothing new. An artificial surface often made anteriorly or posteriorly, is called a bite plane. Its purpose is to prevent the jaws from fully closing when the teeth of the opposing arch are brought together for occlusion [4, 5]. This causes the bite to be considered elevated. They are positioned in the front or back of the mouth relative to the maxillary or mandibular arches and their removal or fixation is dependent on the specific treatment need [6, 7, 8-9]. Appetites were the initial inspiration for the bite plate design. By disarticulating the back teeth and deprogram the masticatory muscles, these appliances make it possible to erupt, extrude and straighten the back teeth [5]. However, in order to achieve the appropriate tooth motions, removable bite plates mostly depend on the cooperation of the patient and need periodic modifications. Their drawbacks include the fact that patients may swallow them, experience choking and have them damaged or misplaced easily [10]. In addition to increasing the likelihood of dental caries, they may cause plaque to build up in previously unclean parts of the teeth [11, 12]. To overcome patient compliance and comfort, the designs were modified to directly fix to the teeth as an attachment, which was considered to be a more compliant and convenient form of design. “The most commonly used orthodontic auxiliaries are often described as 'biteplates', 'bite planes', "disarticulators", 'bite blocks', 'bite turbos', "bite ramps", "bite bumpers", "occlusal build-ups" and 'bite raisers' [8]. Over the years several bite planes starting from the first inclined plane described by John hunter in 1771 which was fabricated on the lower anterior teeth to correct a lingually blocked out tooth; maxillary bite planes described by Henry Clay Quinby AND W.G. Bonwill for deep bite correction; maxillary bite plane introduced by Norman Kingsley to jump the bite to Fixed bite turbos fabricated with glass ionomer cement (GIC) or composite resin; or metal fixed bite raisers described by Alsheikho HO & Jomah D [13]; temporary bite raising crowns, semifixed bite blocks like hygienic bite blocks, rubberized posterior bite blocks, we have come a long way [14]. Therefore, it is of interest to review and give an insight into the recent literature updates on various methods of disarticulation in clinical orthodontic practice.

## Indications of disarticulation:

[1] Protection

a. Brackets from debonding

b. Teeth from attrition due to the use of ceramic brackets as suggested by Årtun J 1997 [14]

[2] Malocclusions exhibiting

a. Deepbite

b. Openbite

c. Crossbite

d. Scissorbite

[3] To unlock occlusion for antero-posterior or transverse correction

[4] Temporomandibular disorders

[5] Anchorage reinforcement

[6] Stopping functional shift (due to premature contact)

[7] Occlusal cant and asymmetry.

[8] Trauma from occlusion

## Complications of disarticulation:

[1] Discomfort (eating/speech problems)

[2] Traumatic occlusion, periodontal problem, mobility of teeth

[3] Wear and breakages

[4] Loss of vitality

[5] Undesired intrusion/extrusion

[6] Occlusal cant

[7] Bruxism

[8] Functional shift

[9] Temporomandibular disorder symptoms.

[10] Soft tissue irritation"

## Anterior bite raisers:

When disocclusion and changes to the back teeth are needed, or when disengaging the back contact is needed to fix a posterior crossbite or maxillary expansion, or in cases of a deep bite caused by infra-eruption of the back teeth and a decrease in lower face height, an anterior bite plane is typically prescribed. Figure 1 (see PDF) shows Classification of bite planes. Figure 2 (see PDF) shows Types of Bite Turbos.

## Ideal requirements of the anterior bite plane:

[1] There shouldn't be any interference between the highway space and the height of the anterior bite plane.

[2] A spacing of two or three millimeters should be present in the back.

[3] The patient may insert their tongue into the space, blocking the eruption of the posteriors, if the clearance exceeds 3 mm.

[4] Pain and damage to the masticatory muscles, TMJ and lower incisors may be caused by high anterior bite planes.

[5] The plane's angle of attack is just as crucial as its height; for optimal results, the bite plane should be perpendicular to the lower incisors' long axis.

[6] Causing intrusion of the incisors.

## Effects of the anterior bite plane:

[1] Molar extrusion, especially the lower first permanent molars

[2] Increased lower facial height

[3] Increase in lower mandibular plane angle

[4] Reduced the masticatory muscle activity,

[5] Help maintain a normal condyle position by strengthening the muscles that support it, which in turn may help keep the condyles in a more stable, compatible and functional posture.

## Hawley anterior bite plate:

This is a standard Hawley appliance with a detachable acrylic platform in the front area, behind the upper incisor teeth, aligned with the occlusal plane. While the teeth are in occlusion, the lower incisor chews down on this plane, creating a space for the back teeth to protrude. How the alveolar bone develops determines this. To prepare the freshly produced bone to endure the forces of chewing, the biting plane is progressively ground away. These bite planes primarily lead to the posterior teeth erupting at various rates, which reduce the depth of bite and the anterior teeth erupting at different rates, which promotes proclination and levels the curve of the bite. As a neuromuscular deprogrammer, this bite plane may alleviate clicking, discomfort and other joint symptoms associated with temporomandibular disorders (TMDs) [11]. Several elevator muscles, including the lateral pterygoid, relax as a result of this joint unloading. In addition, when it comes to posterior dental crossbite, the teeth may be easily corrected with cross elastics since they are not crowded. There needs to be a two to three-millimeter gap at the back and the height of the front bite plane shouldn't obstruct the road. If the space between the back teeth is wider than 3 mm, the patient may block the eruption of their teeth by placing their tongue in the space. However, the masticatory muscles, temporal lobe joints (TMJ) and lower incisors are all susceptible to injury and discomfort when the bite plane is too high in the front. The mandible may also rotate in an undesirable clockwise direction as a result of this. It is preferable if the clinician can elevate the anterior bite plane little by little while they see the bite widening. A further critical component in mitigating undesirable effects is the angle of the plane, which is closely related to height. For optimal force generation and incisor intrusion, a bite plane should be perpendicular to the lower incisors' long axis [12]. All of its revisions, as detailed in the literature that follows, are subject to the same meticulous examination of bite plane height.prevented by extending the acrylic plate to cover the incisal border of the upper teeth [11, 15].

## Sved bite plane:

This bite plane was designed in 1944 to minimize the side effects of proclination of anterior teeth by holding back the forward component of force. Here, the proclination of the anteriors is prevented by extending the acrylic plate to cover the incisal border of the upper teeth [11, 15].

## Bite plate with expansion screw:

For patients with transverse deficit who have deep bite correction throughout the mixed dentition period, a detachable device with an anterior bite plate that incorporates a jackscrew might be helpful [15].

## Bite plate with a reverse inclined plane:

Although it resembles a flat anterior bite plane at first glance, the angle at which it engages the lower incisors and causes the jaw to slide forward is what really makes it unique. As a result, the mandibular teeth are encouraged to shift forward from distal occlusion toward neutral occlusion. To prevent the lower incisors from flaring, the dentist must carefully monitor the occlusion throughout this operation [11]. When a kid is still developing, the reverse-incline appliance may help with minor issues such as deep bite, mandibular retrusion and anterior extrusion. Additionally, it may be used as a retention device after aggressive functional treatment, such as twin block [16].

## Catalan's appliance:

Lower anteriors may have their anterior crossbite corrected with this appliance's slanted plane. When incisors are coming in, they utilize them. Proper occlusion of the lingually emerging incisor is guided by the inclination plane with 45 degrees of angulation. When worn for an extended period of time, the posterior teeth might over-erupt, causing an anterior open bite [17]. The recommended maximum wear length is three weeks. When the back teeth emerge, this device also aids in correcting a deep bite.

## Nociceptive trigeminal inhibition tension suppression system (NTI-TSS):

The anterior bite block is a custom-made, prefabricated appliance that fits over the upper or lower teeth. In instances of migraines, bruxism and temporomandibular disorders (TMDs), it has been used as a deprogrammer [18]. Because it is individualized, the acrylic or thermoplastic material that is molded into the device's base and adjusted in tandem with the teeth guarantees a snug fit. When the patient moves their jaw, the NTI is adjusted such that their back teeth do not touch. Although it is most often worn at night, there are also daytime versions available.

## Anterior bite turbos:

## Metal turbos:

Ormco Corporation created a lingual upper incisor bracket in 1994 and it was only a basic modification of that [13]. The idea behind it was that metal, as opposed to the pliable acrylic used in traditional bite planes, would be more effective in deprogramming the muscles. The design was straightforward, basic and long-lasting. However, lisping and unpleasant tooth vibrations were also issues and the lingual architecture of the top incisors might vary, making placement challenging at times [10].

## Anterior resin turbos:

The introduction of resin turbos was a response to the problems with metal turbos. Turbos may be 3D-printed, made on-site using direct or indirect methods, or constructed using materials like acrylic gels, band cement, bracket adhesives and lingual retainer glue [19]. The lingual aspect of the upper central incisors is the typical location for anterior resin turbos. Patients with low angles should have them. You should use care when using them in high-angle instances to prevent the danger of unwanted posterior extrusion, but they may still be administered. For occlusal load distribution, resin turbos are often attached to central incisors. Although lateral incisors are not preferred due to their weaker roots, they might be considered as an alternate site if the patient experiences lisping. The turbo would be longer and more incisal if the bite were deeper. The recommended duration of usage for a turbo is 6 months; however, this might vary depending on the severity of the original overbite [20]. Additionally, the periodontal health of the mandibular incisors should be carefully monitored by the doctor. These are similar to palatal composite blocks, which are customized anterior bite blocks with regular composite, have uniform contact of the lower incisors and have the advantage of good patient compliance [21].

## Modified ligature bite plane:

Their shape is customized to match the patient's mouth. Over time, they shrank in size and became more practical, fitting snugly onto patients' teeth for improved comfort and compliance. This change is made after careful evaluation of the height of the biting planes. After making a bite plane and pressing it firmly to the palatal region (behind the incisors) with the ring finger, the bite plane is secured using ligature wire that is tied to the canines and incisors. With this chair-side approach, you can get the job done quickly and easily [22].

## Modified nance palatal button or fixed anterior acrylic bite plate:

Adding a biting plate to the Nance appliance, also known as a palatal acrylic button, is one way to improve it [19]. Because it permits the instantaneous installation of brackets on the lower anterior teeth, this device is priceless as a treatment accelerator. In individuals who experience the premature loss of primary teeth, it may also help to preserve the vertical dimension. This kind of splint is preferable to detachable ones in many instances of TMJ because it prevents the buccal occlusion from becoming too immature and lets the jaw move freely laterally.

## Anterior functional bite turbos:

Turbos that have been beveled to fix the crossbite are known as functional turbos. The lingual surfaces of two or more lower incisors are beveled after resin turbo material is bonded to their incisal edges. When the teeth make contact with the beveled surface, it moves the lower jaw backward and pushes the upper incisors forward [10]. A modest anterior crossbite is the most prevalent condition that they are used to treat [21].

## Posterior bite raisers:

To fix an anterior crossbite, a posterior bite plane opens up space for the front teeth. As a result of the vertical intrusive pressures acting on the back segments, they have also found utility in treating open bites. Reducing lower facial height is achieved by the intrusion of posterior teeth, correction of an anterior open bite and anticlockwise rotation of the jaw.

## Ideal requirement of posterior bite planes:

[1] should be hygienic

[2] minimum bulkiness

[3] less interference with speech

[4] less intrusive to tongue space

## Removable posterior bite plane:

## Hawley posterior bite plane:

Hawley posterior bite plane is an occlusal appliance designed to cover the posterior teeth while maintaining the mandible in an open position, approximately 3-4 mm beyond its physiologic rest position. Advantages include a reduction in posterior dentoalveolar height, relative protrusion of front teeth and an upward and forward rotation of the jaw due to the application of basically vertical pressures [11]. This is useful for fixing an open bite. To achieve the necessary movement of the teeth in situations of scissor bite, an inclined plane or ramp might be used. In detachable appliance treatment, it is used for crossbite correction alongside the Z spring. The tooth tips labially when the Z spring is activated.

## Disadvantages of a removable posterior bite plane:

[1] Must be worn nearly full-time

[2] Patient cooperation is problematic

[3] Possibility of breakage while out of the mouth

[4] Bulky

[5] Uncomfortable for the pt

[6] Interference with tongue movements

[7] Ill-fitting ones may cause mucosal trauma

## Fixed posterior bite plane:

## Posterior resin turbos:

Typically, the occlusal surfaces of the mandibular first molars, particularly the supporting cusps, are treated with posterior resin turbos [10]. These are shown in circumstances when the average angle is present. The mandibular second deciduous molars are an appropriate location for these in preteens and adolescents. During fabrication, the only problem that arises is isolation. Some physicians choose to implant them on maxillary teeth to avoid this issue and provide greater isolation. An additional layer of bracket retention may be achieved by extending resin pads over the bracket pad. Preparing mandibular molars with deep intercuspation with composite alone may avoid anchoring loss [23].

## Bonded posterior bite plane with expansion screw:

Hyperdivergent individuals often have their maxillary cross bites corrected using expansion screws, such as hyrax. Bonded posterior bite planes disarticulate to allow unrestricted extension of the posteriors, although long-term storage causes molar intrusion. For optimal results, lift the bite 3 to 4 mm over highway space. For cases of maxillary constriction, a posterior bite plane maxillary expansion appliance may help disarticulate the jaw from its locked position and promote maxillary expansion. For the same reasons, this works in unilateral posterior cross-bite as well as bilateral [10].

## Spring-loaded posterior bite blocks:

To correct an open bite that is caused by the poking out of the teeth, one might consider using this device. Woodside and Linder-Aronson explained the spring-loaded block in 1986 [24]. The types of acrylic material that encase the back of the teeth possess helical springs comprising 0.9 millimeters of high-strength stainless-steel wire that were implanted in the acrylic encasing the teeth. There is force delivery which is affected through helix activation. The bite blocks can be bilaterally linked using acrylic plates and 1 mm stainless steel wires. These may be lengthened till reaching up to the cingulum or simply covered over the incisors to prevent them from becoming untied. These neuro-muscular extend their utility in periodic intervals, besides the pressures that the masticatory muscles are subjected to. Due to its shape, it can also be used as an instrument that assists a person in quitting bad habits. Being a form of a distraction gesture, this appliance involves the patient having to press actively on his jaw to close it [25].

## Rapid molar intruder (RMI):

If you have an open bite, this device may help with the active incursion of your molars by attaching it to your upper and lower teeth. To treat open bites in patients who refused treatment, Carano first employed it. Mould bands hold the elastic modules and coil springs together. The teeth are subjected to an initial force of 800g while they are in occlusion, which decreases to 450g at the end of the first week and 250g at the end of the second week. Because the RMI force line is laterally aligned with the molars' center of resistance, one potential side effect of the treatment is the crown tilting to the buccal region; however, this may be mitigated by using the lingual and transpalatal arches. Using bite blocks instead of molar bands and modifying RMI provides the additional benefit of spreading the force of RMI across the block rather than focusing it exclusively on the molars [26, 27].

## Magnetic bite blocks:

This device is designed to fix an open bite by utilizing magnets that resist each other in opposite arches. The incursion of posterior teeth is caused by their repelling action. To prevent reactions with saliva, samarium cobalt magnets, which are the most common, must be enclosed in the mouth. When it came to fixing an open bite, Dellinger was the pioneer in using magnets. The MAD IV device, which came later, made use of stainless steel-coated neodymium iron boron magnets, which are three times stronger than samarium-cobalt magnets. The lateral and vertical shearing forces are both supplied by this apparatus [28].

## Magnetic bite blocks:

This device is designed to fix an open bite by utilizing magnets that resist each other in opposite arches. The incursion of posterior teeth is caused by their repelling action. To prevent reactions with saliva, samarium cobalt magnets, which are the most common, must be enclosed in the mouth. When it came to fixing an open bite, Dellinger was the pioneer in using magnets. The MAD IV device, which came later, made use of stainless steel-coated neodymium iron boron magnets, which are three times stronger than samarium-cobalt magnets. The lateral and vertical shearing forces are both supplied by this apparatus [28].

## Implant-supported bite blocks:

A more modern method for inserting back teeth is the use of posterior bite blocks in conjunction with skeletal anchoring. The combination of posterior bite blocks, springs or elastics and TADs creates a compound effect that enables stable en masse intrusion in patients who are not growing. But incisions are used to insert the implant, making it an intrusive method. An auxiliary device to avoid buccal health issues is the transpalatal arch. To make attaching the elastics a breeze, the acrylic blocks have hooks built right in [29].

## Guray bite raiser:

This is a custom-made, short-term bite raiser. It is used with ligature wire or elastomer to secure it after being placed into the helmet tubes and fitted over the occlusal surface of the molars. It comes in two sizes, one for kids and one for grown-ups. Because it is prefabricated, it requires less time to put in and is easier on the patient's back [30, 31].

## Temporary bite raising crowns:

One set of molars gets self-cure acrylic, while the other set gets GIC cement. Be cautious to guarantee correct occlusal contact on both sides during manufacturing. Their use aids in the treatment of frontal crossbite. Being less cumbersome, more comfortable and causing less speech impediment, they are superior to any detachable bite raiser [32].

## Posterior functional bite turbos:

For minor dental crossbites, beveled turbos might be used. The beveled occluding surfaces of these turbos, which are also called functional turbos, direct the opposing teeth to line up in the correct spot. For Class II patients, they may also be used on premolars to enhance disarticulation. Just like a bonded Twin Block appliance, these turbos work by disarticulating and repositioning the mandible while the occluding premolars move over the beveled surfaces. Similarly, patients using aligners might have empty rectangular attachments placed on the aligners' occlusal surfaces to achieve a similar result [10].

## GIC bite block:

## Most commonly used method for disarticulation. It has some drawbacks:

[1] Difficulty in chewing as a single point of contact

[2] Damage to enamel on removal.

[3] GIC splint removal is cumbersome & time-consuming

[4] Supra eruption of adjacent teeth as well as opposing teeth

## GIC splint remover:

To overcome the shortcomings of the GIC block, a GIC split remover has been developed. By using this ligature wire, two loops (bigger and smaller) are made, connecting each other. Larger loop adapted to the occlusal surface & smaller loop to the buccal surface of 1st or second molars. Stabilize the lager loop with GIC blocks. After the desired correction is achieved, hook an instrument through the small loop and pull to remove the splint from the tooth (E). The GIC-wire assembly generally comes away in a single piece; any cement remaining on the occlusal surface can easily be removed with an ultrasonic scaler [33].

## Semi-fixed bite raiser:

Inserted into the auxiliary or main molar tube, this hygienic disoccluding device consists of two zigzag loops 2 mm wide constructed of 017x025 ss wire that occlude from the first premolars to the first molars on both sides [34].

## Modified posterior bite block:

Acrylic bite block with holes on the occlusal surface of the blocks for the removal of excess cement and added retention [35].

## M block for bite opening:

A triangle is formed with ss wire, edge pointing towards the cusp tip, with a 3 mm diameter helix for ease of holding the block for insertion in the tube, then luting with GIC [36].

## Hygienic bite blocks:

The placement of the bite plane, anterior or posterior, depends on the mandibular plane angle. Stainless steel band material is used with perforations and the Sleeve is welded onto the band material. A sleeve with 0.7 mm stainless steel wire attached and then the Bite block was fabricated. Bite block secured into lingual sheath. It can be used along with fixed appliance mechanotherapy, is easy to fabricate and has less chairside time with no adhesive remnants [37].

## A bite block - new technique [38]

It"s a full-time appliance, hygienic and has good patient cooperation.

## Rubberized posterior bite block: An effective fixed temporary bite opening tool adhering to COVID-19 protocols:

Using high-speed air-rotor handpieces to remove GIC bite blocks exposed clinicians to excessive risk of saliva, blood and aerosol contamination during the COVID-19 pandemic, making their use completely contraindicated. A novel, easy-to-use and cost-effective solution to these problems is the Fixed Rubberized Bite Block, which is made by tying ligature wires reinforced with rubber sleeves from the molar tube to the lingual sheath of the same molar band. This design crosses over the occlusal surface of the molar and addresses these drawbacks [39].

## Semifixed posterior acrylic bite blocks:

This is a semifixed bite block that has two components: a buccal component, T-shaped, made with 21 gauge and a lingual component, + shaped, made with 23 gauge. Two lingual sheaths, one facing buccally and the other vertically, are fused. Wire components for the buccal and lingual regions were put into their prescribed slots. We acrylicize bite blocks. Reliability, convenience and unilateral dosing of this bite block are all top priorities [40].

## Posterior bite block with ligature wire:

Posterior bite blocks, which are cemented with GIC, most of the time, the patient reports loose bite blocks and debonded brackets. To overcome this, acrylic blocks are made with embedded metal tubes, which allow insertion of ligature wire and the block is secured around the molars, lingual to labial [41].

## Conclusion:

The use of bite raisers has become more popular in recent years as an essential aid in treating some of the most challenging cases, which necessitate bite opening for furtherance of orthodontic therapy. A number of factors, including the patient's facial structure, the degree of skeletal malocclusion, the initial degree of deep bite, the angle of the mandibular plane and the mechanics of the planned therapy, are considered while deciding which bite raiser to use. A thorough understanding of the mechanics, amount, thickness, position and duration of bite raiser application is very critical to avoid any inadvertent effects on the overall treatment outcome.

## References

[1] Pativetpinyo D (2018). J Appl Oral Sci..

[2] Roy AS (2013). Int J Orthod Milwaukee..

[3] https://old.bansdoc.gov.bd/sub-3/150contemporary/150Contemporary%20Orthodontics.pdf.

[4] Civen OI (1945). Am J Orthod Oral Surg..

[5] https://www.academia.edu/.

[6] Philippe J (1996). J Clin Orthod..

[7] Madsen R (1998). J Clin Orthod..

[8] Jackson S, Sandler PJ (1996). J Clin Orthod..

[9] Banks PA, Carmichael G (1998). J Clin Orthod..

[10] Kravitz ND (2018). J Clin Orthod..

[11] https://shop.elsevier.com/.

[12] Batoni G (2001). Eur J Oral Sci..

[13] Alsheikho HO, Jomah D (2021). J Indian Orthod Soc..

[14] Årtun J (1997). Eur J Orthod..

[15] Singh G (2015). Jaypee Brothers Medical Publishers..

[16] Emami MS (2007). J Indian Soc Pedod Prev Dent..

[17] Tiwari N (2020). Acta Sci Dent Sci..

[18] Stapelmann H, Türp JC (2008). BMC Oral Health..

[19] Pratiwi D, Purwanegara MK (2020). Sci Dent J..

[20] https://www.ortodoncia.ws/.

[21] Pamukçu H, Özsoy OP (2021). Angle Orthod..

[22] Krishna M (2022). Acta Sci Dent Sci..

[23] Retrouvey JM (1996). Am J Orthod Dentofacial Orthop..

[24] Woodside DG, Linder-Aronson S (1991). Am J Orthod Dentofacial Orthop..

[25] Doshi UH, Bhad WA (2011). Am J Orthod Dentofacial Orthop..

[26] Cinsar A (2007). Angle Orthod..

[27] Carano A (2005). Am J Orthod Dentofacial Orthop..

[28] Dellinger EL (1986). Am J Orthod..

[29] Doshi UH, Bhad WA (2011). J Clin Orthod..

[30] Güray E (1999). J Clin Orthod..

[31] Ceen RF (2002). J Clin Orthod..

[32] Vibhute PJ (2006). J Clin Orthod..

[33] Surendra L (2013). J Clin Orthod..

[34] Sharma VK (2016). J Clin Orthod..

[35] Beane RA-Jr (1999). Semin Orthod..

[36] Gupta M (2016). APOS Trends Orthod..

[37] Maheshwara VV (2016). APOS Trends Orthod..

[38] Sidna A (2017). Adv Dent Oral Health..

[39] Palathra CG (2021). J Contemp Orthod..

[40] Chachada A, Pedgaonkar S (2020). Orthod J Nepal..

[41] Ahamed N (2014). J Indian Orthod Soc..

